# Study of ecosystem service functions in typical receiving areas of the South-to-North Water Diversion Central Route based on a set of long time series

**DOI:** 10.1371/journal.pone.0302588

**Published:** 2024-05-15

**Authors:** Shaolei Guo, Shifeng Sun, Xianqi Zhang, Yang Yang, Yupeng Zheng

**Affiliations:** 1 School of Water Conservancy, North China University of Water Resources and Electric Power, Zhengzhou, China; 2 Collaborative Innovation Center of Water Resources Efficient Utilization and Protection Engineering, Zhengzhou, China; 3 Technology Research Center of Water Conservancy and Marine Traffic Engineering, Zhengzhou, Henan Province, China; University of Ferrara, ITALY

## Abstract

Hebi is located in the northern part of China’s Henan Province and is a typical receiving area for China’s South-to-North Water Diversion Project. The assessment of habitat quality and water yield over a long time series is important for evaluating the stability of ecosystem services in Hebi and other receiving areas and for maintaining ecological security and promoting sustainable development. This paper aims to evaluate and dynamically analyse habitat quality and water yield in Hebi, and analyses the characteristics of changes in spatial and temporal patterns of land cover types, habitat quality and water yield in Hebi over the past 20 years, using 2000, 2005, 2010, 2015 and 2020 as horizontal years. The results indicate that: (1) During the study period, the overall land use type in Hebi City has been constantly changing, with the most significant conversion from arable land to other land types; combined with its landscape pattern index, Hebi City has a general characteristic of significant landscape fragmentation and complexity in land use. (2) Habitat quality in Hebi shows an overall trend towards better development, with water availability decreasing and then increasing; the zoning of ecosystem services in Hebi is divided into three classes: superior, good and general, with the area covered by the superior and general classes expanding year by year. (3) Correlation analysis by SPSS software shows that the correlation between habitat quality and landscape pattern index is greater than the correlation between habitat quality and climate change. Additionally, the correlation between water availability and climate change is greater than the correlation between water availability and landscape pattern index.

## 1 Introduction

Natural ecosystems maintain the dynamic balance of the Earth’s living systems and ecological environment by providing services such as ecosystem goods and ecosystem functions that guarantee the sustainable development of human societies and ecosystems. In recent years, research on ecosystem service functions has become increasingly available and has been applied in policies such as the zoning of ecological functions at the national level, subordinate administrative regions, and various watersheds at multiple scales [1–3], the delineation of ecological protection red lines [4, 5], ecological compensation [6–8], and the assessment of the carrying capacity of resources and the environment [9, 10]. Timely and accurate assessment of ecosystems is vital to human productive life through sensible grooming and monitoring of ecosystems. As urbanisation accelerates, especially in the context of China’s new phase of major infrastructure development, the country’s ecosystems are under serious threat. China’s modernisation process has been seriously affected by a variety of ecological crises brought about by the degradation of ecosystems, such as increased soil erosion, frequent flooding and accelerated species extinction [11].

There are many approaches to the study of ecosystems, and Costanza in 1997 proposed 17 classifications of ecosystem services and used these 17 ecosystem service functions to evaluate ESV (Ecosystem service value) globally [12]; Ouyang et al. followed Costanza’s research progress and made a preliminary assessment of the value of ecosystem services in China using the shadow price method and alternative engineering method [13], demonstrating that terrestrial ecosystems in China contain huge economic benefits; Based on the research of foreign scholars [14, 15], Xie et al. reduced ecosystem service functions into 9 categories and revised the value equivalent factor of ecosystem service assessment in combination with China’s actual situation [16], laying a foundation for the research in China’s ecological field. Other ecosystem assessment methods, such as the energy value assessment method, the value quantity assessment method and the object quality assessment method, are also widely used in various fields in China [17–19]. In recent years, with the development of computer technology and the emergence of remote sensing, GIS and other spatial technologies, there has been an increasing amount of research into the application of spatial data models, and the study of ecosystem function has begun to shift to a model-based approach [20]. A number of models for assessing ecosystem services have emerged internationally, such as InVEST model developed by Stanford University, MIMES model developed by the University of Vermont, UFORE model developed by the Forest Service of the United States Department of Agriculture among many other, through the simplification of the algorithm, using the "big data" analysis technology based on spatial modeling and geographic information system. These models are applicable at the global scale, watershed scale or landscape scale and are highly generalisable [21]. Of these, the InVEST model is the most mature and has been often used both nationally and internationally. In 2011, Polasky et al. used the InVEST model to quantify ecosystem service functions and biodiversity in Minnesota from 1992 to 2001 [22]; In 2016, Redhead et al. modelled the annual water yield of 22 UK catchments using the InVEST model and compared the model outputs with river flow data measured in the UK National River Flow Archive, showing that:relatively simple models can accurately measure ecosystem services [23]; In 2016, Guo et al. used the CLUE-S and InVEST models to analyse the impact of land use change on water-producing ecosystem functions in the study area over the last 25 years based on land use scenarios, using the South Four Lakes watershed in China as the study area [24].

At the end of 2014, the middle route of China’s South-to-North Water Diversion project was officially commissioned. With the massive transfer of water from the South-North Water Transfer Project, the ecology of the rivers and lakes along the route has continued to recover, the water environment has improved, and the groundwater levels in the cities along the route have rebounded, making the receiving areas along the South-North Water Transfer Project of great research value. However, by combing through previous studies related to the South-North Water Transfer, most of them focus on the water source area of the South-North Water Transfer [25–28], and few studies have been conducted on its receiving area. The city of Hebi is a typical receiving area of the South-North Water Transfer Central Line. Since the transfer of water began at the end of 2014, as of 2019, the South-North Water Transfer Project has transferred a total of 250 million cubic metres of water to Hebi. Benefiting from the South-North Water Transfer Project, Hebi’s water resources have been greatly replenished and the city’s ecosystem has been restored. This paper takes Hebi city as the research object, obtains Landsat satellite remote sensing images through remote sensing technology, and processes the satellite images to obtain the land use data of the three phases before and two phases after the opening of the water supply. With the support of platforms like ENVI, ArcGIS, and InVEST, we assessed the habitat quality and water availability in Hebi from 2000 to 2020. We explored their spatial and temporal characteristics in depth to study the changes in ecosystem service functions resulting from the commissioning of the South-North Water Transfer Central Project. The results of the study can provide a theoretical basis and scientific reference for assessing and post-evaluating the ecosystem service functions of other large inter-basin water transfer projects. Additionally, it can offer strategic support for the rational use of water resources, ecological protection, and economic and social development in other receiving areas of the South-North Water Transfer Project.

## 2 Study area

Hebi is located in the northern part of Henan Province, China, with a total area of 2,182 square kilometres. Climatically, Hebi has a warm temperate monsoon climate with an extremely uneven spatial and temporal distribution of precipitation. In 2021, summer precipitation alone will account for more than 80% of the annual precipitation, and precipitation is mainly distributed in the central and western regions, but less in the east. In terms of water resources, an analysis of the water resources bulletin for the past ten years shows that the total annual average water resources in Hebi is 361 million cubic metres, with a per capita water possession of 200 cubic metres, which is less than one tenth of the national per capita possession, and is a typical arid and water-scarce region.

**Fig 1:**
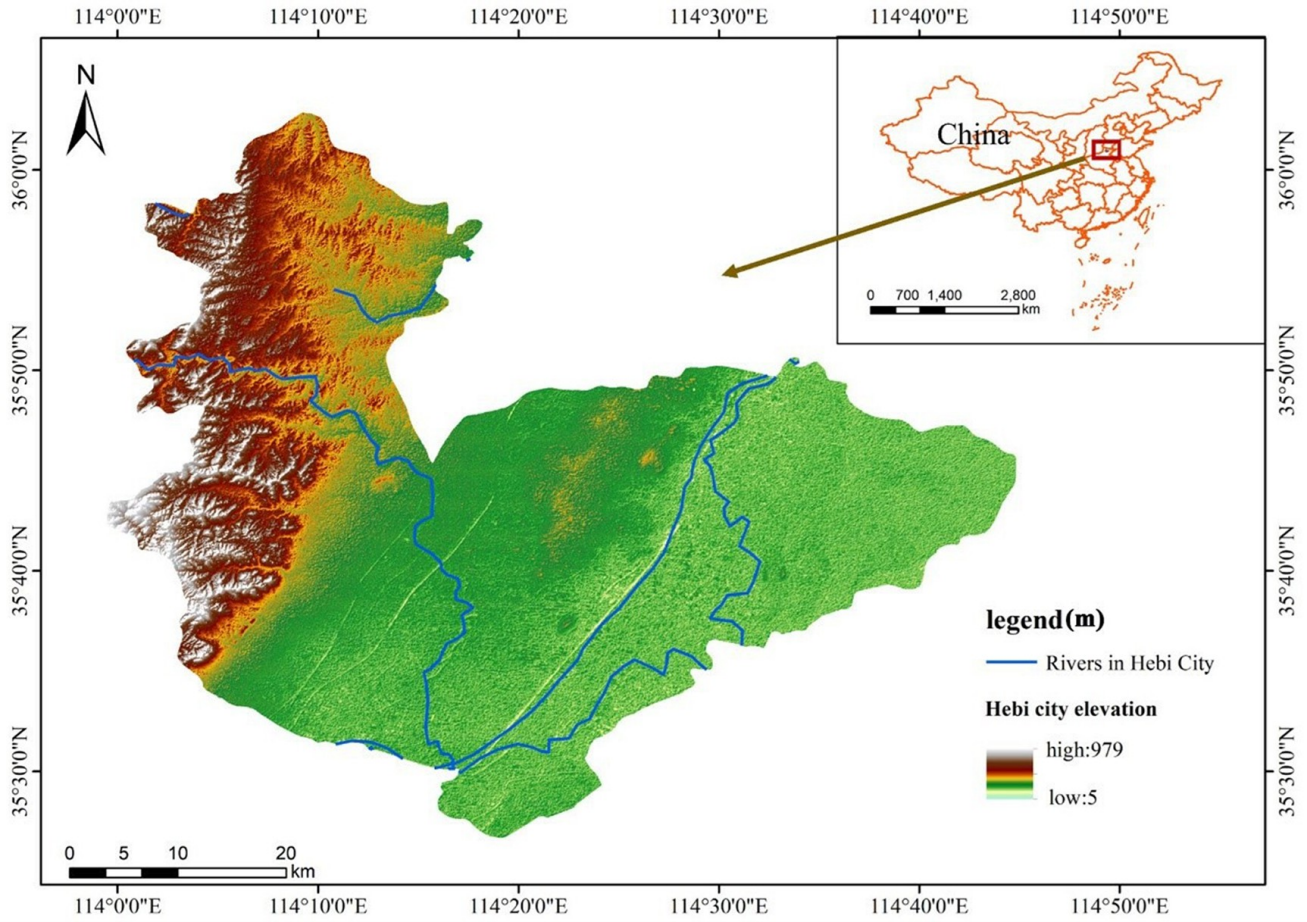
Geographical location of Hebi city.

As one of the receiving areas of the South-North Water Transfer Central Project, the South-North Water Transfer Central Project in Hebi City begins at the Canghe Canal inverted siphon outlet inflow embankment and ends at the border of Tangyin County, with a total length of 30.8km. Compared with other receiving areas, Hebi has more serious problems in terms of water shortage, and the ecological and economic benefits of South-North water transfer are more obvious. Comparing the geographical and climatic conditions of the receiving areas, most of them are partly mountainous, while the rest of the area is mostly plain, and all of them are characterised by uneven spatial and temporal distribution of precipitation, which is more typical of the city of Hebi. In general, Hebi has characteristics common to most receiving areas and is in the middle of the main canal of the South-North Water Transfer Central Project, so it can be studied as a typical receiving area for the South-North Water Transfer Central Line. The specific geographical location of Hebi is shown in Fig 1, and this figure is created using ArcMap 10.7, URL:wwwarcgis.com. DEM elevation data from Geospatial Data Cloud (http://www.gscloud.cn/).

## 3 Materials and methods

**Fig 2:**
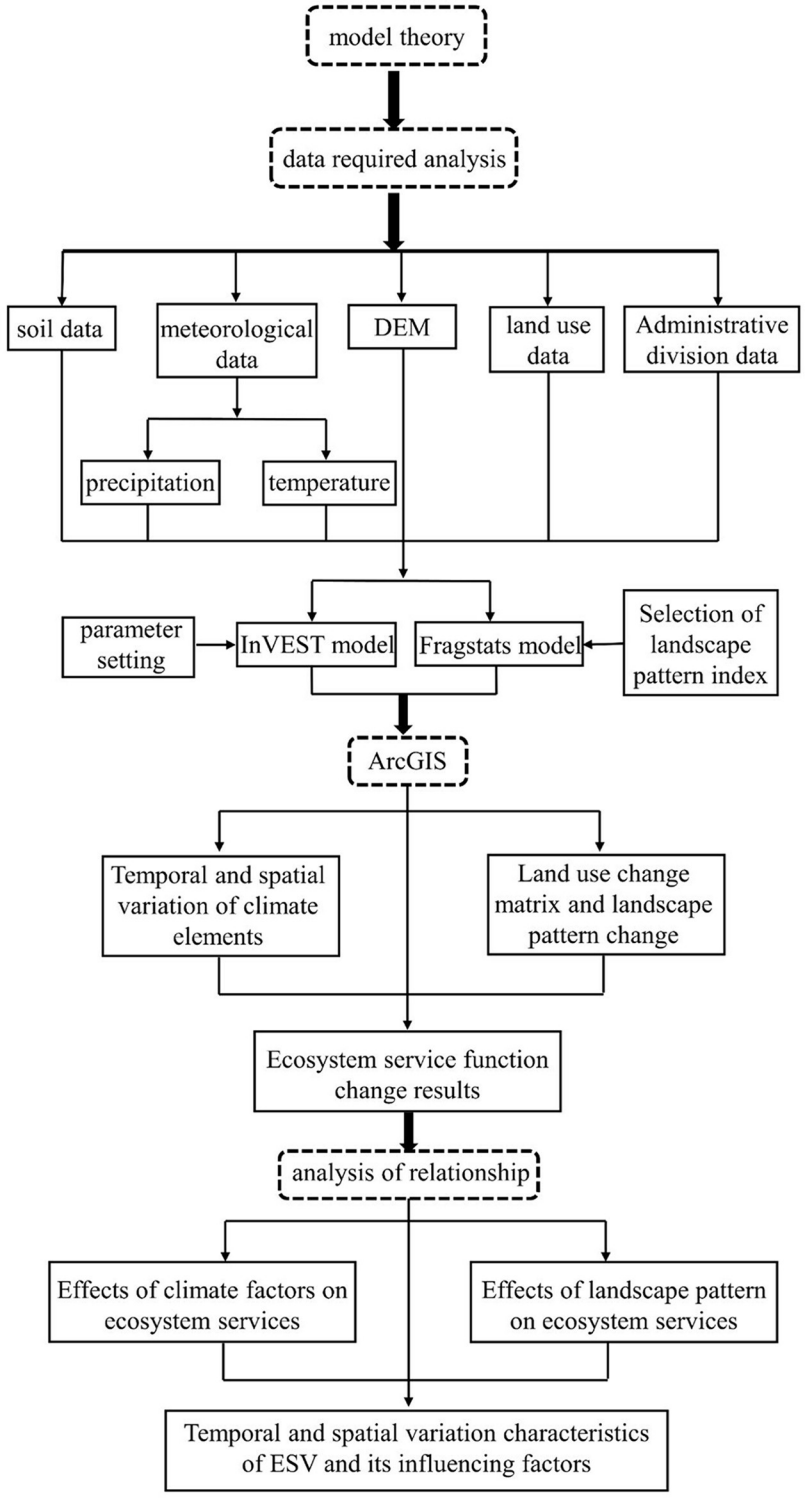
Research technology roadmap.

In this study, Hebi City, a typical water demand area of the middle route of the South-to-North Water Diversion project, As a city located in the Yellow River Basin, Hebi City is facing increasing pressure and challenges to its ecosystem. The Habitat Quality Module is able to comprehensively consider the landscape pattern, vegetation cover and biodiversity of different habitat types to more fully assess the ecological quality of Hebi City’s various regions. By assessing habitat quality, we can fully understand the health of ecosystems in Hebi City and provide a scientific basis for ecological environmental protection and restoration. Meanwhile, Hebi City is a typical receiving area of the South-to-North Water Diversion Center Line, and the Water Yield module can assess the water availability capacity and hydrological processes in different areas, which can help to understand the distribution of water resources, the supply situation, and the degree of satisfaction of urban water supply and ecological demand. By evaluating water yield, it can provide a scientific basis for water resource management, ecological environmental protection and disaster risk management in Hebi City, and promote the sustainable utilization of urban water resources and the healthy development of the ecosystem. was taken as the research area, and the habitat quality and water yield of Hebi city were evaluated by ArcGIS, ENVI, InVEST and other platforms, so as to study the changes in ecosystem service functions brought by the middle route of the South-to-North Water Diversion project before and after its water supply. Correlation analysis between natural conditions and ecosystem service functions was carried out by SPSS 25 software to investigate the correlation between ecosystem service functions and natural conditions, and to reveal the impact of landscape pattern indices and climate change on ecosystem service functions. The specific research route is shown in Fig 2.

### 3.1 Data sources and processing

#### 3.1.1 Land use data

**Fig 3:**
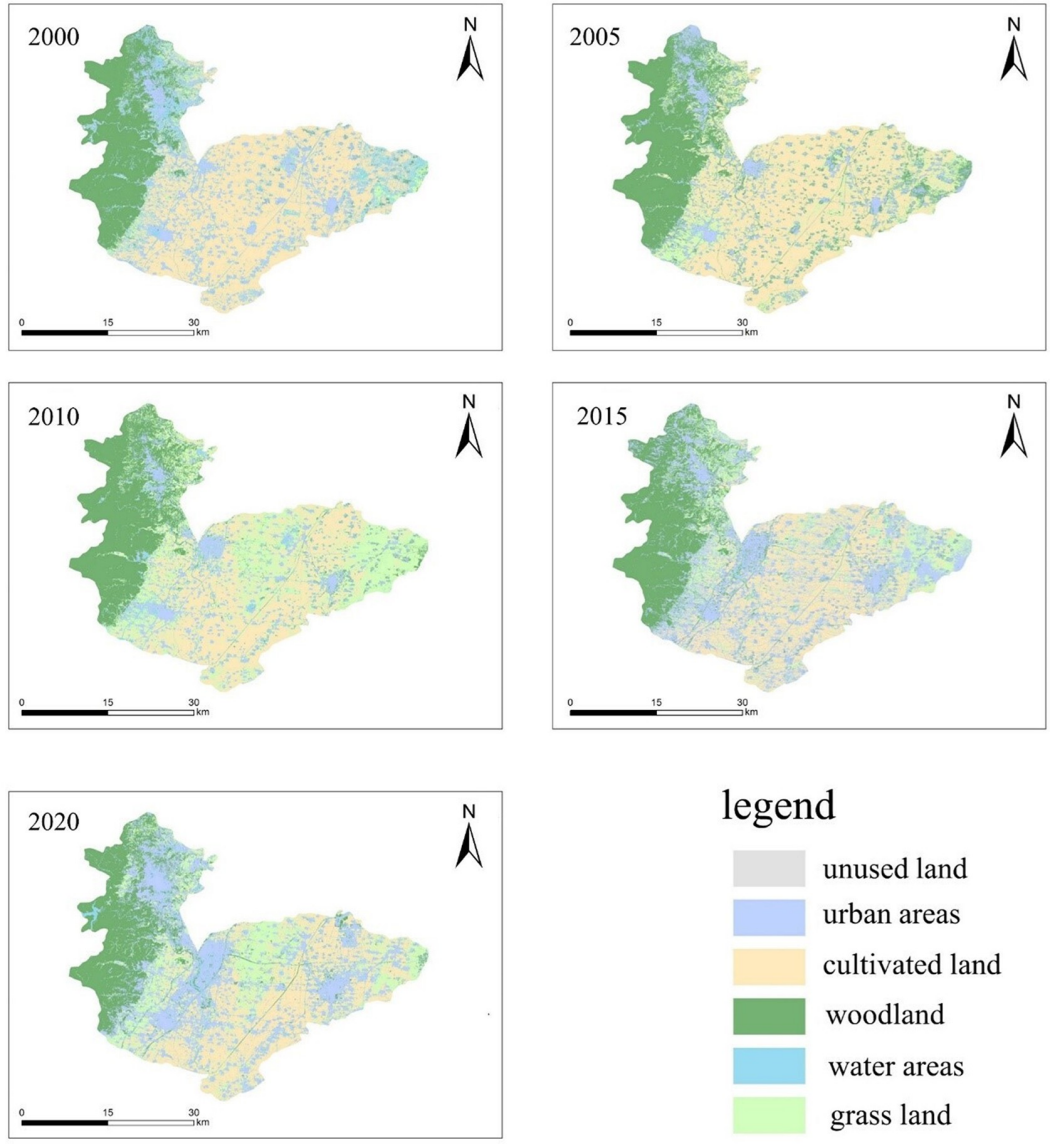
Distribution of land use types in Hebi City from 2000 to 2020.

This study is based on Landsat4-5 TM and Landsat8 OLI-TIRS remote sensing image data of five periods in 2000, 2005, 2010, 2015 and 2020 provided by Geospatial data cloud (http://www.gscloud.cn/). By means of the Environment for Visualizing Images (ENVI) platform, the satellite remote sensing images were processed with radiometric calibration and atmospheric correction tools. According to the vector file of the administrative area of Hebi City, data transformation, clipping and image enhancement were carried out on the processed satellite remote sensing images, and finally five phases of remote sensing images before and after the water supply of the Middle Route of the South-to-North Water Diversion Project of Hebi City were obtained. After Kappa coefficient test and multiple field investigations, Kappa coefficients of satellite remote sensing interpretation data in the 5 phase were 0.79, 0.82, 0.85, 0.83 and 0.89, respectively, which met the requirements of this study. In this paper, the MORTRAN model is used for atmospheric calibration, and the ENVI5.1 toolbox FLAASH atmospheric calibration model is used. The FLAASH atmospheric calibration model does not need the measurement data when imaging the remote sensing image, and it has high accuracy, which can effectively eliminate the interference of the atmosphere on the imaging of the remote sensing image. Radiometric calibration was performed by selecting the remote sensing image of the study area acquired by the Radiometric Calibration Tool in the ENVI5.1 toolbox. According to "National Standard (Classification of Land Use Status) GB/T 2010-2017" and the actual land use situation of Hebi City, the land use types in the study area were divided into six categories: urban areas, cultivated land, grass land, woodland, water areas and unused land. The distribution diagram of land use types in Hebi City from 2000 to 2020 is shown in Fig 3, and this figure is created using ArcMap 10.7, URL:wwwarcgis.com.

From Fig 3, we can see the land use changes in Hebi City from 2000 to 2020: the area of grassland, water area and cropland decreased significantly, forest land and unutilized land showed a slow decreasing trend, and urban land showed a continuous increasing trend.

#### 3.1.2 Habitat quality

Land use raster data for the study area from 2000-2020 has been prepared in section 3.1.1. In this study, according to the geographical environment and land use landscape pattern of Hebi city, referring to the manual of using InVEST model and related studies [29], towns and cultivated land disturbed by strong human activities were selected as threat factors, and the information of their maximum threat distance and weight decayability are shown in Table 1.

**Table 1:** Threat factors and their associated attributes.

THREAT	MAX_DIST	WEIGHT	DECAY
urbanization	5	1	exponential
farming	1	0.6	linear

When preparing the threat source data, the urban and arable land in the land use data from 2000 to 2020 were extracted separately and the threat factor was assigned a value of 1, while the rest were assigned a value of 0.

Habitat suitability indicates the strength of an organism’s ability to survive and reproduce in that habitat and can be interpreted as a habitat score for each land use type with values between 0 and 1. 0 and 1 are used when sufficient information on the habitat characteristics of the common species is not available, with 1 being habitat and 0 being non-habitat. Sensitivity, on the other hand, indicates how responsive a habitat type is to a threat source, again with values ranging from 0 to 1, with values closer to 0 indicating poor sensitivity. Based on currently available references [30], and taking into account the actual situation of land use and habitat threat distribution in Hebi, the sensitivity of each habitat type to threats was determined as shown in Table 2.

**Table 2:** Sensitivity of different habitat types to threats.

Type of land use	Habitat suitability	Sensitivity	
		urbanization	farming
urban areas	0	0	0
cultivated land	0	0	0
woodland	1	0.5	0.8
water areas	1	0.4	0.2
grassland	1	0.8	0.6
unused land	0	0	0

From the results, it is easy to see that all habitats have different sensitivities to different threat sources, with grassland having the highest sensitivity to urbanization and water areas having the lowest sensitivity to urbanization; woodland having the highest sensitivity to farming and water areas having the lowest sensitivity to farming. Woodland is more susceptible to farming, while water areas and grassland are more sensitive than farming to projects such as urbanization.

When setting the half-saturation and parameter *k*, first set k to 0.5 in each simulation, and then reset the value of *k* according to the highest degraded grid value.

#### 3.1.3 Water output

The water yield module requires eight necessary data: maximum root depth of the soil, annual precipitation, Plant Available Water Fraction, average annual potential evapotranspiration, current land use data, catchment/administrative area, table of biophysical coefficients, and seasonal constant Z.

Data of the maximum root depth of the soil in the study area were obtained from the World Soil Database (HWSD) soil dataset provided by the National Tibetan Plateau Scientific Data Center (http://www.tpdc.ac.cn/zh-hans/). The data were downloaded and transferred to the ArcGIS platform for processing to obtain the maximum root depth of the soil in the Hebi area. The maximum root depth of the soil in Hebi were all located in the 0-100 cm range.

**Fig 4:**
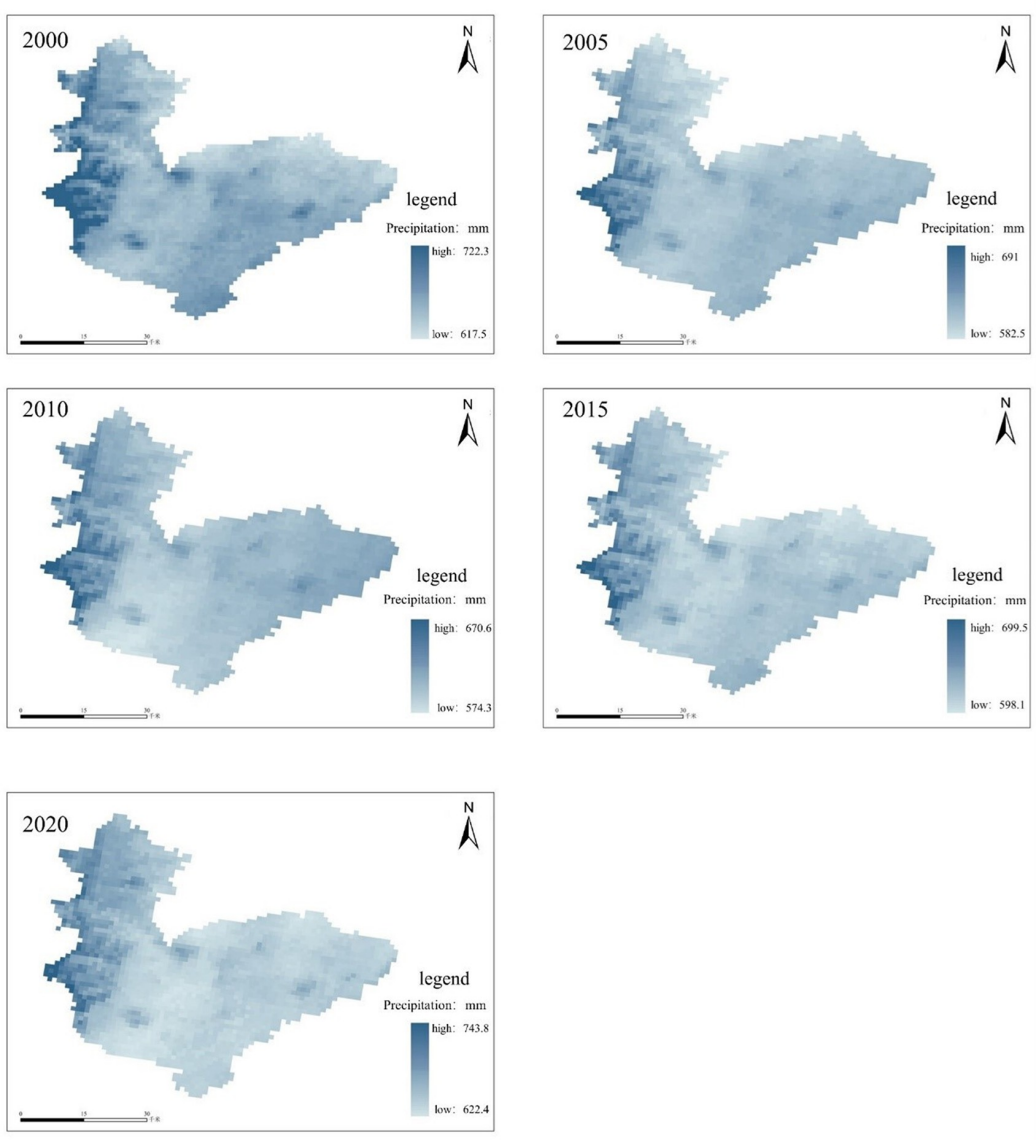
Annual precipitation of Hebi City from 2000 to 2020.

Annual precipitation data are derived from the 1km resolution monthly precipitation data set for China from 1901 to 2021 provided by the National Earth System Science Data Center (http://www.geodata.cn/). Data were preprocessed and extracted month by month through ArcGIS platform, and then monthly data were accumulated to form the annual data required by this research. The annual precipitation data of Hebi from 2000 to 2020 is shown in Fig 4, and this figure is created using ArcMap 10.7, URL:wwwarcgis.com.

The basic data of Plant Available Water Fraction is also from the National Tibetan Plateau Scientific Data Center. Based on the sand content, clay content, powder content and organic carbon content provided in the obtained data, the available plant water content is calculated using Formula (1).


$$\begin{array}{l} PAWC=54.509-0.132T_{sand}-0.003{\left(T_{sand}\right)}^{2}\\ -0.055T_{silt}-0.006{\left(T_{silt}\right)}^{2}-0.738T_{clay}+0.007{\left(T_{clay}\right)}^{2}\\ -2.688T_{oc}+0.501{\left(T_{oc}\right)}^{2} \end{array}$$ (1)

The annual average potential evapotranspiration data in this study were obtained from CGIAR-CSI, the Consultative Group on International Agricultural Research of the Spatial Information Consortium (https://cgiarcsi.community/). The biophysical coefficients table contains mainly the root depths and vegetation evapotranspiration coefficients (Kc) of the corresponding plants, and the InVEST model links the corresponding data into the model by means of the ground class code for calculation. After observing previous studies [31] and the InVEST model use manual, and taking into account the actual situation in Hebi. The biophysical coefficients for Hebi City were derived and tabulated in Table 3 below.

**Table 3:** Table of biophysical coefficients in Hebi.

Description	Land type number	root_depth	Vegetation evapotranspiration coefficient(Kc)	Vegetation cover
urban areas	1	10	0.2	0
cultivated land	24	1500	0.865	1
woodland	48	3000	1.1	1
water areas	63	10	1.015	0
grassland	89	2600	1.055	1
unused land	99	200	0.3	0

According to the InVEST model operating manual, the seasonal factor Z takes on a range of values from 1 to 30. In this study, the seasonal constants were examined in relation to the actual situation in Hebi and the data published in the Hebi Water Resources Bulletin, and it was found that when the seasonal constant Z was 20.25, the resulting water yield was closest to the actual value. In this study, the seasonal constant Z was taken as 20.25.

### 3.2 research method

#### 3.2.1 InVEST model habitat quality and water yield module

High quality habitats are usually relatively intact, and habitat quality is defined as the ability of an ecosystem to provide conditions suitable for the survival of individuals and populations based on the availability of subsistence resources, the number of organisms reproducing and present, and is also considered a continuous variable in the model, ranging from low to medium to high [32]. The Habitat Quality module of the InVEST model combines the status of the land use type, the parameters related to the threat factors and the degree of habitat response to the threat factors to produce a Habitat Quality Index and a Habitat Degradation Index for the study area. The specific calculation method and the principle of the formula for the habitat quality module refer to the study by Song et al. [33].

The Annual Water Yield module of the InVEST model is based on the assumption of coupled hydrothermal equilibrium, i.e. the actual evapotranspiration is subtracted from the precipitation at each grid to obtain the water yield at each grid. Limited by the length of the article, the specific principle and calculation formula can be referred to the study of Redhead et al. [23].

#### 3.2.2 Landscape pattern index analysis

The mainstream method of land use landscape pattern research is the landscape pattern index analysis, which can reflect the spatial structure of different land use types more accurately [34]. The quantitative presentation of different land use patterns using figures allows for a more accurate study of landscape pattern changes, while we consider the impact and performance of local ecosystem functions and the sensitivity of landscape indicators, this paper will select six type-level landscape pattern indices and eight landscape-level landscape pattern indices to assess the landscape pattern indices in Hebi, and the specific calculation indicators are shown in Table 4.

**Table 4:** The landscape pattern index selected for this study.

category	landscape pattern index	abbreviation	level	implication
area	the largest patch index	LPI	Type / Landscape	Percentage of the largest patch of a type / landscape in the total area
density	patch numbers	NP	Type / Landscape	The sum of the number of patches of all patch types in a certain type / landscape
	patch density	PD	Type / Landscape	The density of a certain type of patch in a certain type / landscape
edge	marginal density	ED	Type / Landscape	The boundary length per unit area of a certain type / landscape patch in the unit is studied.
shape	landscape shape index	LSI	Type / Landscape	The ratio of the total length of all patch boundaries of a type / landscape in a unit to the theoretical equivalent minimum total length is studied.
assembling and parting	degree of polymerization	AI	Type / Landscape	Reflect the aggregation degree of patches in a certain type / landscape
	the degree of spread	CONTAG	Landscape	Reflect the spread of different landscapes
diversity	shannon diversity index	SHDI	Landscape	It reflects the heterogeneity, richness and complexity of landscape.

#### 3.2.3 analysis of relationship

The current statistical analysis software SPSS25 has been widely used in correlation analysis studies, and its Pearson correlation coefficient can be used to correlate the relationship between each ecosystem service function with the landscape pattern index and climate change, thus reflecting the intrinsic link between natural climatic factors and the landscape pattern index and ecosystem service functions in the study area [35]. A two-sided test was used to test the significance of the correlations in the study results, and indicators whose significance was less than 0.05 and 0.01 were taken as reference indicators.

#### 3.2.4 Land use transfer matrix

A land use transfer matrix is a matrix that uses two-dimensional matrices for the study of ordered pairs of real numbers. The land use matrix allows for a more vivid visualisation of land use change in the study area between two points in time to understand the overall characteristics of land use change. Its calculation formula is as follows:

$$S_{ij}=[\begin{array}{cccc} S_{11} & S_{12} & \cdots & S_{1n}\\ S_{21} & S_{22} & \cdots & S_{2n}\\ \vdots & \vdots & \vdots & \vdots \\ S_{m2} & S_{m3} & \cdots & S_{mn} \end{array}]$$ (2)

Where: *S_ij_* indicates the use of land from time point T_1_ to time point T_2_; *m* and *n* are both land use types.

Ethical Approval:This paper does not contain any studies with human participants or animals performed by any of the authors.

## 4 Results and analysis

Based on the above, we analyze the land use, landscape pattern, ecosystem services, ecosystem services, ecosystem services, and drivers of ecosystem services, respectively.

### 4.1 Analysis of land use change results

**4.1.1 Temporal land use change characteristics.** Based on the processing of land use data in Chapter 3, the calculation was collated by ArcGIS software and Excel software, and the percentage of land use types in Hebi for different years from 2000 to 2020 is shown in Table 5.

**Table 5:** Percentage of land use types in Hebi in different years.

year		urban areas	cultivated land	woodland	water areas	grassland	unused land
2000	Number of grids	297108	1006101	543745	273507	192863	68376
	proportion	12.47%	42.24%	22.83%	11.48%	8.10%	2.87%
2005	Number of grids	289484	896971	713598	96096	319303	66248
	proportion	12.15%	37.66%	29.96%	4.03%	13.41%	2.78%
2010	Number of grids	267658	695257	620192	104100	635472	59021
	proportion	11.24%	29.19%	26.04%	4.37%	26.68%	2.48%
2015	Number of grids	467182	688599	585340	115200	469198	56181
	proportion	19.62%	28.91%	24.58%	4.84%	19.70%	2.36%
2020	Number of grids	658604	615434	506431	121994	438911	40326
	proportion	27.65%	25.84%	21.26%	5.12%	18.43%	1.69%

**Fig 5:**
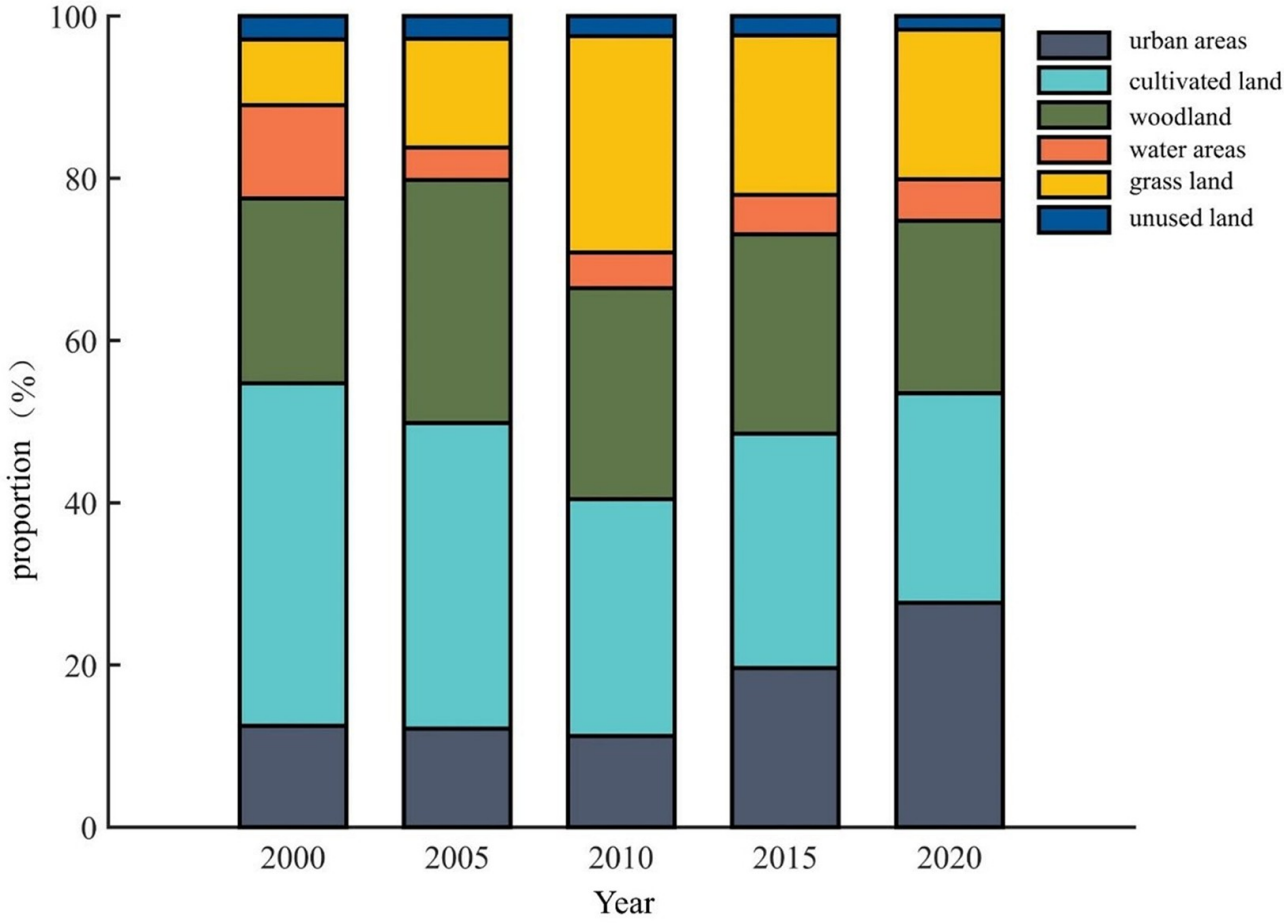
Land use type percentage stacking map of Hebi City.

As can be seen from Table 6, with rapid urbanisation over the two decades, the proportion of urban land classes in Hebi grew from an initial 12.47% to 27.65% in 2020, the largest increase; a gradual reduction in the proportion of cultivated land, from an initial 42.24% in 2000 to 25.84% in 2020, the only land category to have declined significantly in two decades; The proportion of woodland has fluctuated without significant change over the past two decades, and at 21.26 per cent in 2020, it is essentially the same as in 2000; the water area has been on a downward trend since 2000, but with the national emphasis on water resources in recent years and the implementation of inter-basin water transfer projects such as the South-North Water Transfer Central Project, the percentage of water areas in Hebi has regained more than 5% in 2020, fully reflecting the ecological benefits of the South-North Water Transfer Central Project; grassland has also seen a large increase over the last two decades, with the proportion of 2020 grassland area increasing by 10.33% compared to 8.10% in 2000; the proportion of unused land itself is relatively small and steadily declining in size. It is not difficult to see from the changes in the above data that with the continuous improvement of China’s emphasis on ecological environment construction in recent years, the implementation of major projects such as the south-to-north water diversion project, river diversion to supplement Hanjiang river and national water network, and the implementation of policies such as returning farmland to forest, closing mountains and greening, the cultivated land has decreased significantly, while the grassland area and water area have been improved to some extent. The temporal variation of land use type is shown in Fig 5.

**Table 6:** Land use transfer matrix of Hebi City, 2000-2005(km^2^).

		2005					
	land type	grass land	urban areas	cultivated land	woodland	water areas	unused land
2000	grass land	85.80	9.75	7.90	17.18	2.94	0.04
	urban areas	10.76	153.04	3.45	74.10	6.05	0.04
	cultivated land	97.65	12.68	745.91	19.44	10.82	0.01
	woodland	10.61	35.57	1.31	419.91	1.97	0.02
	water areas	15.33	9.49	7.71	1.61	25.55	0.02
	unused land	0.01	0.01	0.06	0.02	0.02	0.05

#### 4.1.2 Characteristics of land use spatial change

Using the land use transfer matrix method, based on ArcGIS10.7 software for processing and analysis, through data statistics, the land use transfer from 2000 to 2020 is obtained, as shown in Tables 6–9 below.

**Table 7:** Land use transfer matrix of Hebi City, 2005-2010(km^2^).

		2010					
	land type	grass land	urban areas	cultivated land	woodland	water areas	unused land
2005	grass land	136.75	14.55	53.74	26.74	5.60	0.05
	urban areas	28.05	161.27	12.33	21.26	5.62	0.01
	cultivated land	62.69	18.50	717.55	14.14	3.40	0.04
	woodland	18.44	14.49	7.98	395.89	16.01	0.04
	water areas	19.12	6.07	4.13	8.15	9.63	0.04
	unused land	0.02	0.02	0.01	0.03	0.03	0.04

**Table 8:** Land use transfer matrix of Hebi City, 2010-2015(km^2^).

		2015					
	land type	grass land	urban areas	cultivated land	woodland	water areas	unused land
2010	grass land	192.23	45.16	42.73	43.52	2.40	0.03
	urban areas	18.71	313.26	14.59	27.01	4.32	0.03
	cultivated land	35.52	66.00	423.18	10.65	2.10	0.01
	woodland	33.46	40.82	8.88	330.42	6.30	0.03
	water areas	12.06	6.23	3.63	15.20	5.57	0.03
	unused land	0.05	0.00	0.01	0.04	0.02	0.01

**Table 9:** Land use transfer matrix of Hebi City, 2015-2020(km^2^).

		2020					
	land type	grass land	urban areas	cultivated land	woodland	water areas	unused land
2015	grass land	119.54	41.78	41.52	30.62	19.11	0.03
	urban areas	25.68	388.67	24.43	15.37	21.32	0.01
	cultivated land	59.22	27.44	421.35	5.82	18.46	0.02
	woodland	30.94	44.53	2.85	348.47	17.52	0.05
	water areas	0.14	0.62	0.03	0.51	9.38	0.02
	unused land	0.04	0.03	0.01	0.01	0.05	0.04

As seen Tables 6–9, the land types with more frequent land conversion during the study period were cultivated land, woodland, grassland and urban areas. From 2000 to 2005, the transfer area of cultivated land was the largest, mainly converted into grassland and woodland; followed by urban areas, mainly converted to woodland. From 2005 to 2010, the transfer area of grassland was the largest, mainly converted into cultivated land and woodland; followed by cultivated land, mainly converted to grassland and woodland. From 2010 to 2015, the transfer area of cultivated land was the largest, mainly converted into urban areas and grassland; followed by grassland, mainly converted to urban areas, cultivated land and woodland. From 2015 to 2020, the transfer area of grassland was the largest, mainly converted into urban areas, cultivated land and woodland; followed by cultivated land, mainly converted to grassland and urban areas. The woodland in the study area is mainly located on the mountains in the west, the rest is distributed around the arable land and grassland, and the grassland is distributed adjacent to the cultivated land, so the interchange between the three land types of cultivated land, woodland and grassland is more frequent. After 2010, China’s urbanization accelerated, and the transfer of urban land increased rapidly. Overall, cultivated land, woodland, grassland and urban areas were the more frequently converted land categories during the study period. This is closely related to national policies and social development, such as the national policy of returning farmland to forests, which largely influences the conversion of cultivated land to woodland and grassland; and the rapid development of urbanization in recent years, which has prompted the conversion of other land to urban areas. The analysis of the whole land class conversion in Hebi City during the study period is consistent with the development of Hebi City in the past 20 years, indicating that the result of land classification is relatively accurate.

### 4.2 Analysis of the results of the change in landscape pattern

Using the land use raster data obtained in Chapter 3.1 and Fragstats 4.2 software, eight indicators at two levels were screened to analyse the landscape pattern of the study area from 2000-2020. Due to the small area of unused land, it may cause outliers in the calculation of landscape pattern, so the result of unused land is not considered in the analysis of landscape pattern.

#### 4.2.1 Changes in class-level landscape pattern indices

**Fig 6:**
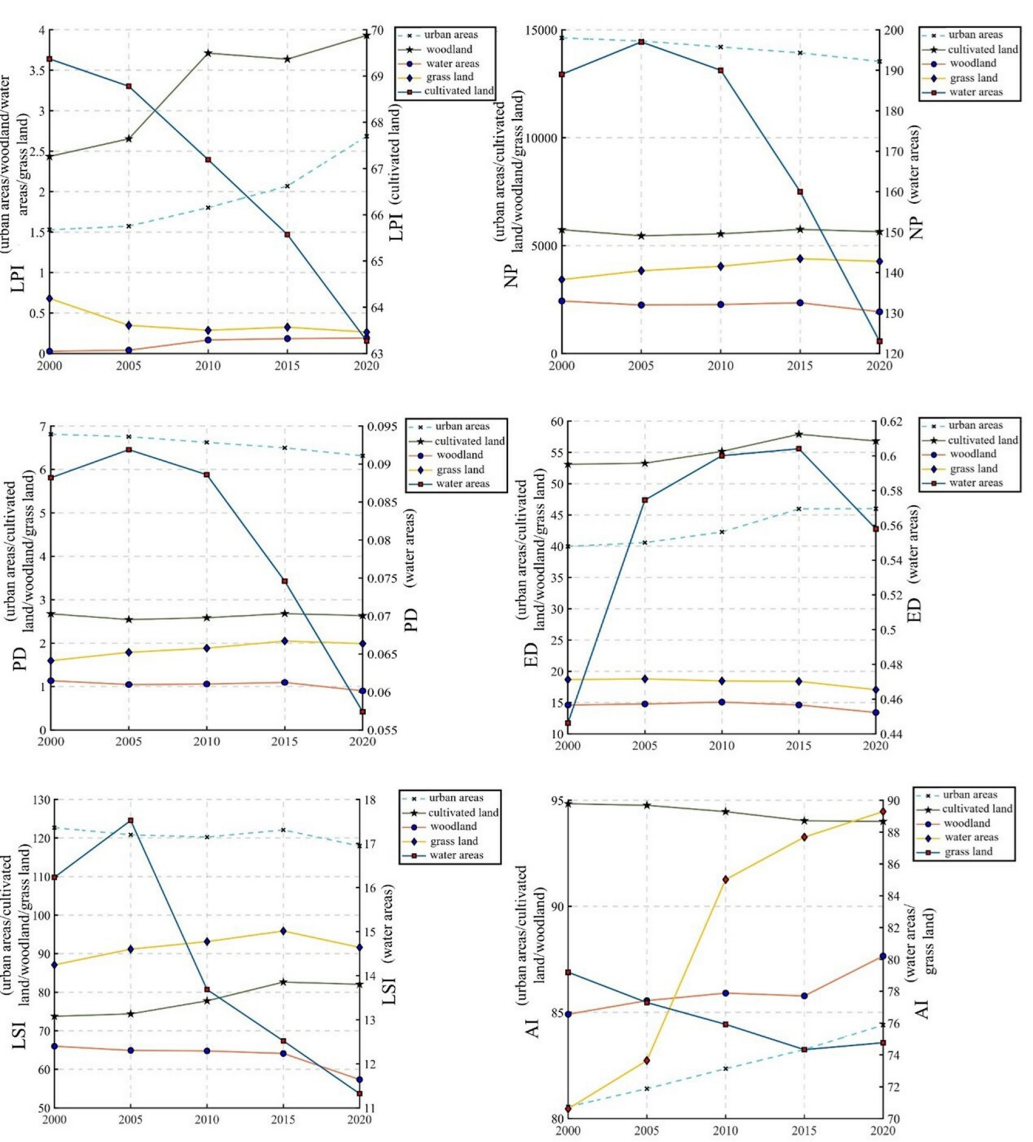
Trend of landscape pattern index at type level in Hebi City.

The type level landscape pattern index was calculated, and the result was presented as the change trend of different land use types in different years of a single index, as shown in Fig 6.

The analysis shows that the maximum number of patches (LPI) index shows that the area of cultivated land, although decreasing from year to year, has a clear advantage over other land types; there is a clear upward trend in woodland and urban areas, while water and grassland are more stable. Observing the number of patches (NP), the water has the lowest number of patches, even though the maximum value taken in 2005 was still less than 200, indicating a less fragmented landscape and a clear downward trend in the NP values of the water during the study period; compared to the waters, the overall NP values of the other land types do not fluctuate much. Looking at patch density (PD), it maintains essentially the same trend as the number of patches (NP) and has the greatest fragmentation in urban areas and the least fragmentation in waters. Looking at edge density (ED), waters still maintain the lowest edge density and show an increasing and then decreasing trend over the study period; the edge density index for cultivated land is consistently the highest of the five landscape categories; the edge density index for urban areas has consistently increased over the last two decades; and the edge density trends for grassland and woodland are largely the same, with less overall fluctuation. Looking at the Landscape Shape Index (LSI), in the 2000-2020 Hebi landscape shape index, urban > grassland > cultivated land > woodland > water; looking at the degree of aggregation (AI), the degree of aggregation and landscape connectivity between urban, woodland and water patches in the study area increased over the 20-year period, while the opposite occurred in the case of cultivated land and grassland, where the degree of aggregation and landscape connectivity decreased to varying degrees. Overall, the trend in the Hebi Landscape Shape Index shows that the landscape shape of the study area has become increasingly regular over the last 20 years.

#### 4.2.2 Changes in landscape-level landscape pattern indices

The results of the landscape level landscape pattern index calculations are shown in Table 10.

**Table 10:** Hebi City 2000-2020 landscape level landscape pattern index statistics table.

	LPI	NP	PD	ED	LSI	AI	CONTAG	SHDI
2000	69.37	26403.00	12.32	63.41	75.40	91.12	62.47	0.87
2005	68.78	26211.00	12.23	64.00	76.07	91.04	61.96	0.89
2010	67.19	26244.00	12.24	65.79	78.15	90.79	60.90	0.91
2015	65.57	26580.00	12.40	68.75	81.57	90.37	59.97	0.93
2020	63.27	25506.00	11.90	66.93	79.47	90.63	59.53	0.96

In terms of landscape levels, there is a general characteristic of significant landscape fragmentation and complexity in Hebi over the period 2000-2020. The Largest patch index (LPI) in the Hebi landscape decreased from 69.37 to 63.27 and the Edge density index (ED) increased from 63.41 to 66.93 over the twenty-year period, indicating a significant degree of landscape fragmentation in the study area; the Number of patches (NP) shows significant volatility, with the maximum value occurring in 2015 at 26580 and the minimum value occurring in 2020 at 25506, with the greatest decrease between 2015 and 2020; Plaque density (PD) has been relatively stable over the last two decades, with a decrease of only 0.42, indicating a slight decrease in spatial fragmentation in Hebi; the Landscape shape index (LSI) has shown a general upward trend over the study timeframe, rising by a total of 4.07 between 2000 and 2020, indicating a trend towards an irregular development of landscape shape and an increase in complexity in the study area; the landscape Aggregation index (AI) has been relatively stable over the last two decades, with a slight decline, indicating a relatively small change in landscape connectivity and a moderating change in heterogeneity within the study area; the Contagion index (CONTAG), which reflects the degree of sprawl of different landscapes, decreases from 62.47 to 59.53 between 2000 and 2020, with a more pronounced downward trend, indicating a decreasing trend in landscape connectivity and an increasing degree of landscape fragmentation in the study area; the Shannon diversity index (SHDI) reflects the heterogeneity, richness and complexity of the landscape. The shannon diversity index in the study area continues to maintain an increasing trend, from 0.87 in 2000 to 0.96 in 2020, indicating that the heterogeneity, richness and complexity of the landscape in Hebi are in an increasing stage.

### 4.3 Ecosystem service function analysis

**Fig 7:**
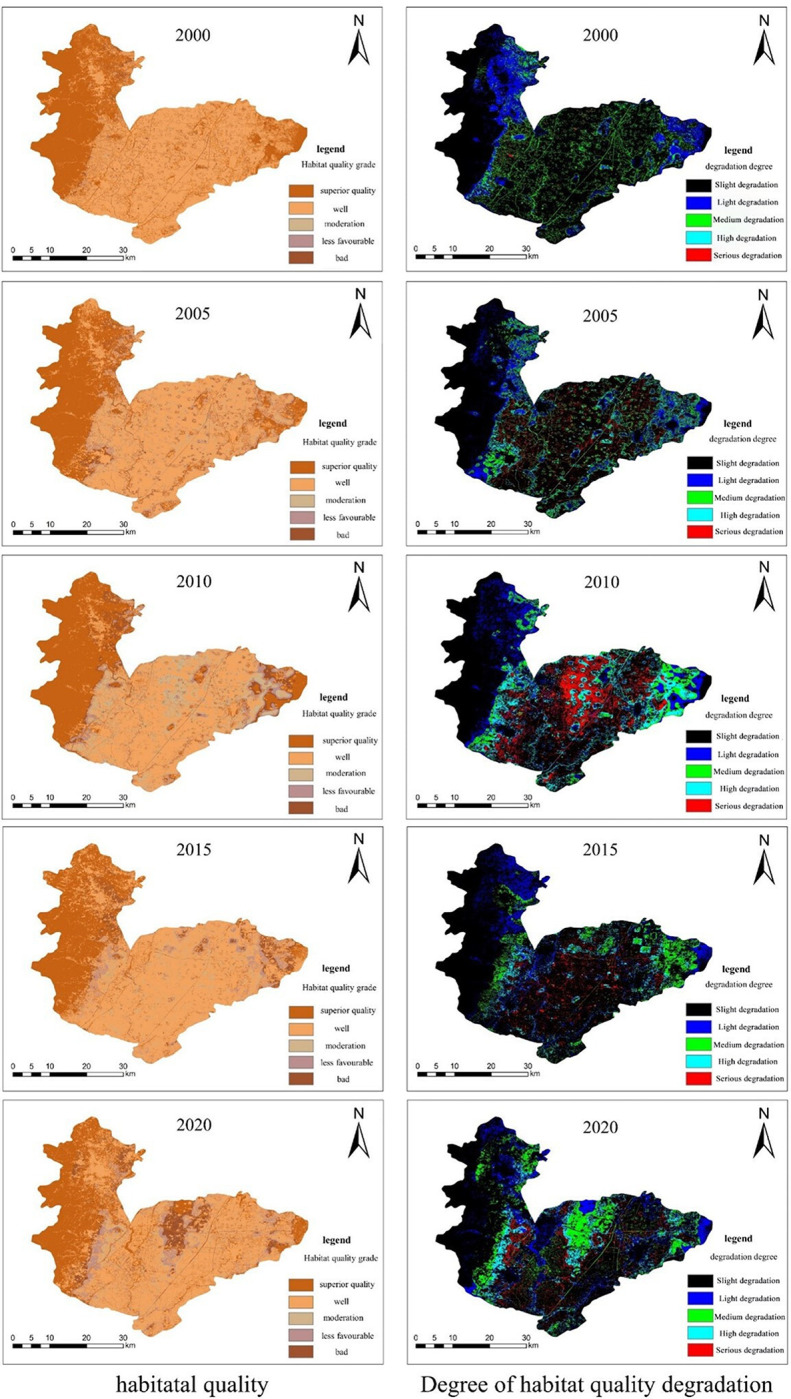
Habitat quality degradation and habitat quality index map for Hebi City over the years.

Based on the land use data and other pre-processed data of Hebi city, the habitat quality module of the InVEST model was used to run to obtain the habitat quality degradation degree map and habitat quality map of Hebi city for five years in 2000, 2005, 2010, 2015 and 2020, and the assessment results were presented as shown in Fig 7 through the functions of raster calculator and reclassification in ArcGIS software, and this figure is created using ArcMap 10.7, URL:wwwarcgis.com.

#### 4.3.1 Spatial and temporal characteristics of habitat quality

The degree of habitat quality degradation in Hebi was classified into five levels of degradation: slightly degraded, lightly degraded, medium degraded, highly degraded and seriously degraded. The extent of habitat quality degradation in Hebi from 2000-2020 is shown in Table 11 below.

**Table 11:** Degree of habitat quality degradation in Hebi City 2000-2020.

		slightly degraded	lightly degraded	medium degraded	highly degraded	seriously degraded
2000	Number of grids	1864607	243820	221820	32116	19337
	Percentage	78.29%	10.24%	9.31%	1.35%	0.81%
2005	Number of grids	1826401	189728	173175	101527	90869
	Percentage	76.68%	7.97%	7.27%	4.26%	3.82%
2010	Number of grids	1565143	247037	164857	194899	209764
	Percentage	65.72%	10.37%	6.92%	8.18%	8.81%
2015	Number of grids	1938997	162710	125429	90280	64284
	Percentage	81.41%	6.83%	5.27%	3.79%	2.70%
2020	Number of grids	1760371	203399	211101	132217	74612
	Percentage	73.91%	8.54%	8.86%	5.55%	3.13%

From the simulation results of the InVEST model, the highest percentage of habitat quality degradation in Hebi during the study period was the slight degradation level, with the proportion basically remaining above 70%, and the overall habitat quality degradation index was relatively small; overall, since 2000, the degree of habitat quality degradation in Hebi has shown a trend of increasing and then fluctuating decline, due to rapid urbanisation and irrational exploitation of natural resources by the economy and society, which led to the highest Habitat Quality Degradation Index in Hebi in 2010. With the implementation of concepts such as ecological protection and high-quality development, the Habitat Quality Degradation Index in Hebi has improved considerably, while the promotion of projects such as the South-North Water Diversion Middle Line Project and the restoration and comprehensive management of the ecosystem in the Pai River Basin in 2014 has led to some improvement in the ecological environment in Hebi, but there is still a gap compared to the early stage of the study.

Spatially, the seriously degraded areas are mainly located in the main urban area in central Hebi and in Jun County in the east and Qixian County in the southwest, and the habitat quality degradation indices in the above areas are all at high levels. After overlaying and intersecting the land use type and habitat quality degradation maps in ArcGIS software, the seriously degraded areas are mostly towns and cities and plots of grassland, woodland and water embedded in cultivated land, and the highly degraded areas are mostly where the cultivated land borders other land types. In the context of strong human activity, there is a greater threat of habitat degradation in towns as well as around cultivated plots, with the eastern part of Hebi consistently maintaining a high quality index of habitat degradation due to its proximity to the urban area of Puyang. Medium degradation is usually found around areas of higher degradation, usually single plots of cultivated land with a small footprint and in more isolated urban areas. Light and slightly degraded areas are generally woodland, grassland and water areas away from urban and cultivated areas and are usually larger and all connected together.

The Habitat quality module of the InVEST model evaluates habitat quality in the study area on a scale of 0 to 1, with values closer to 1 indicating better habitat quality and vice versa. In this study, the habitat quality index was classified into the following five categories: superior, well, moderate, less favourable and bad. The quality index of superior habitats is 0.8-1.0; the quality index of well habitats is 0.6-0.8; the quality index of moderate habitats is 0.4-0.6; the quality index of less favourable habitats and bad habitats are 0.2-0.4 and 0-0.2 in that order. Habitat quality in Hebi from 2000-2020 is shown in Table 12.

**Table 12:** Habitat quality in Hebi 2000-2020.

		superior	well	moderate	less favourable	bad	Raster average
2000	Number of grids	864444	1394190	17025	15410	90631	0.43
	Percentage	36.30%	58.54%	0.71%	0.65%	3.81%	
2005	Number of grids	665852	1316240	79071	132263	188274	0.38
	Percentage	27.96%	55.26%	3.32%	5.55%	7.91%	
2010	Number of grids	641510	1426210	101155	96044	116781	0.38
	Percentage	26.93%	59.88%	4.25%	4.03%	4.90%	
2015	Number of grids	784979	1043252	297994	119158	136317	0.33
	Percentage	32.96%	43.80%	12.51%	5.00%	5.72%	
2020	Number of grids	752222	1303540	111245	86582	128111	0.36
	Percentage	31.58%	54.73%	4.67%	3.64%	5.38%	

From a temporal perspective, the average Habitat Quality Index for Hebi declined steadily between 2000-2015 and did not rebound slightly until 2020. The highest proportion of habitat quality grades is well, followed by superior, indicating that the city of Hebi has a good base of habitat quality. When comparing habitat quality across years, the best year for habitat quality in Hebi was 2000, which not only had the highest average habitat quality index, but also the highest percentage of superior habitat quality, and the lowest percentage of less and bad habitat quality. Since 2000, habitat quality in Hebi has continued to decline until 2015, when it began to recover. Benefiting from the opening of the South-North Water Transfer, the proportion of moderate, less favourable and bad habitat quality has decreased in 2020 compared to 2015, while the proportion of well has begun to rebound.

From a spatial perspective, areas of bad habitat quality are mainly located in the central city of Hebi, with sporadic distribution in the centre and around the eastern arable land and towns and other land types. ArcGIS software overlaps and intersects the land use type and habitat quality, and it is concluded that the habitat quality of Hebi City is superior mainly in the forest land and water area in the western mountainous area and the grassland far away from the urban and cultivated land. Areas with a well habitat quality rating are found in most of the east-central part of the city of Hebi and consistently cover the largest area of the five ratings; areas with moderate, less favourable and bad habitat quality are mostly in central urban areas and large areas of towns, cultivated land, etc. According to the elevation DEM data for Hebi, the higher elevation western undeveloped mountainous areas have the best habitat quality, indicating that the original topography maintains the integrity of the local habitat structure and function better with less disturbance from human activities.

#### 4.3.2 Spatial and temporal variation characteristics of water yield

**Fig 8:**
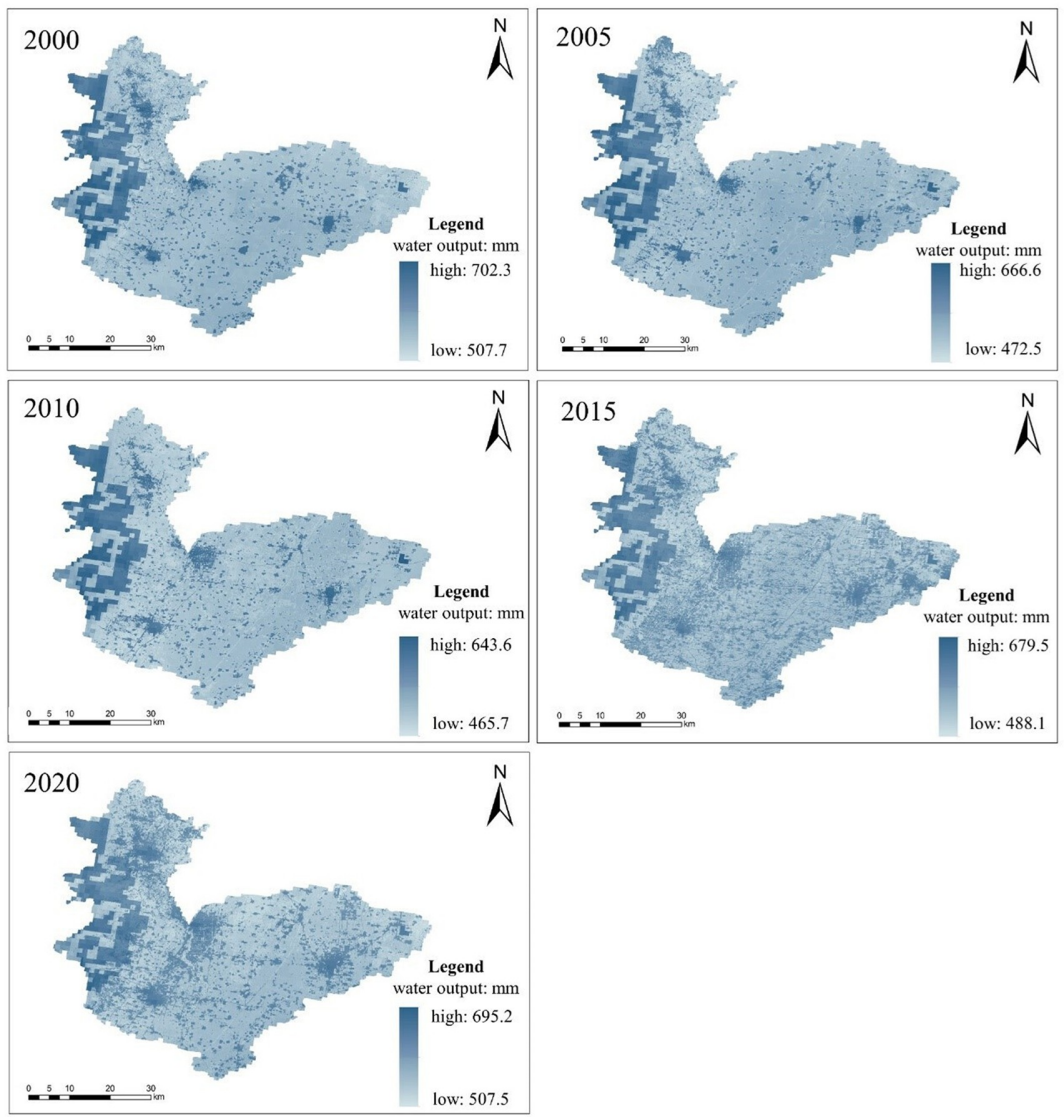
Spatial distribution of water availability in Hebi over the years.

Using the water yield module of the InVEST model, the results were run to obtain a five-year assessment of water yield in Hebi for the years 2000, 2005, 2010, 2015 and 2020, and the results were presented in Fig 8 using the raster calculator and reclassification functions in ArcGIS software, and this figure is created using ArcMap 10.7, URL:wwwarcgis.com.

According to the simulation results of the InVEST model, the total and average water availability in Hebi from 2000 to 2020 shows a decreasing trend followed by an increasing trend. 2010 was the lowest point of water yield, and the maximum water yield, the minimum water yield and the average water yield were the lowest; since then, the water yield has gradually recovered. By the end of the study, 2020, the water yield is basically consistent with the early 2000. The rate of decline in water availability was greatest from 2000-2005, at 5.65%, while the period of highest growth was from 2010-2015, with a rate of 7.29%. The average growth rate over the twenty-year period was 1.57%. The statistical table of water availability in Hebei from 2000-2020 is shown in Table 13.

**Table 13:** Hebi City 2000-2020 water availability statistics table.

year	2000	2005	2010	2015	2020
Total /10^10^mm	1.33	1.26	1.23	1.32	1.35
Average /mm	559.55	527.88	514.91	552.46	568.32

**Fig 9:**
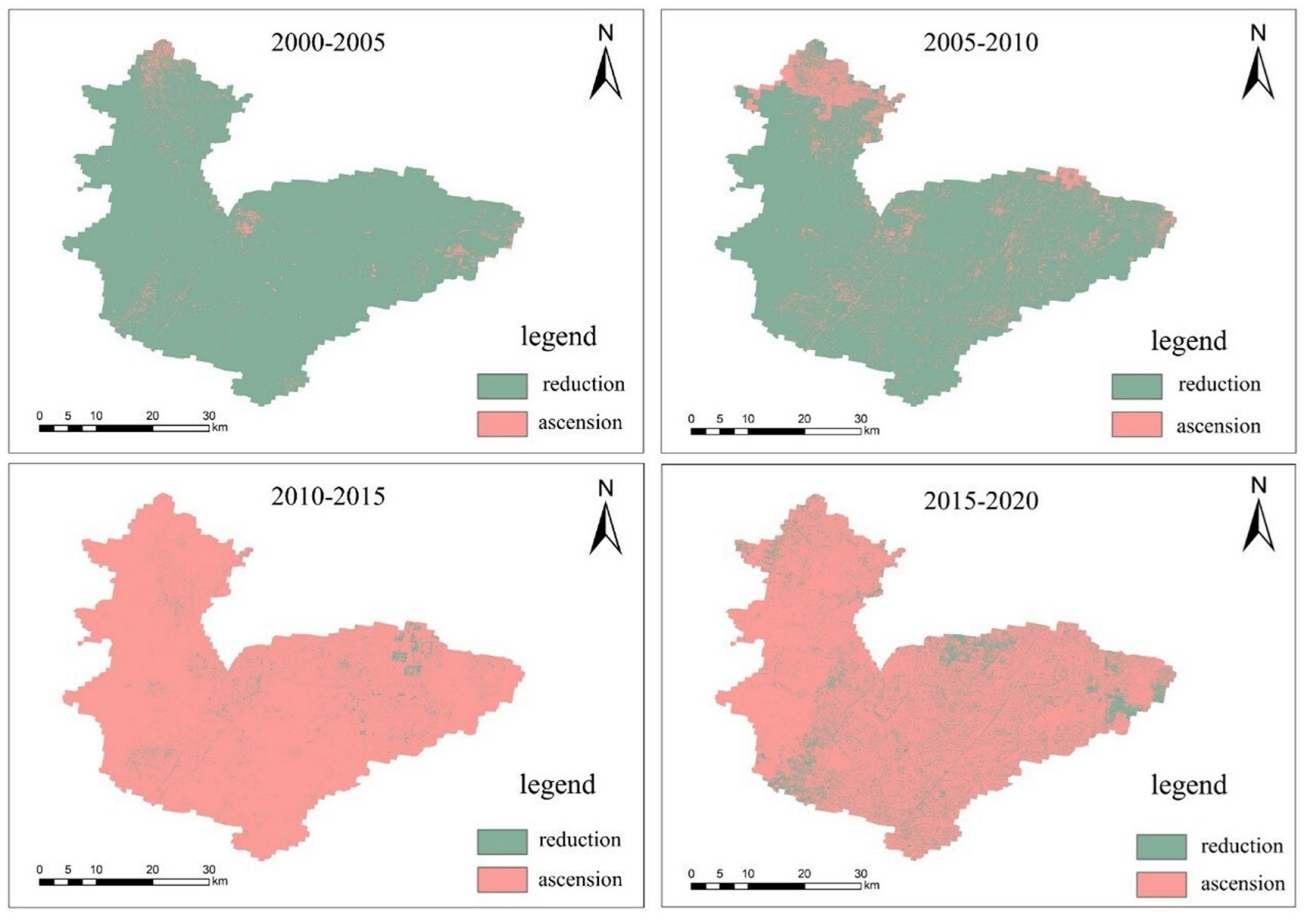
Spatial variation of water availability in Hebi.

As can be seen from Fig 8, the spatial structure layout of water availability in Hebi from 2000 to 2020 remains basically stable, showing an overall distribution characteristic of high in the northwest and low in the southeast. For five consecutive study periods, all maintained the highest water yield in the woodlands of the western mountainous region, with a multi-year average of 590-660 mm; the average water yield in the east-central region was lower, with some areas of high value scattered, for example, the main urban area of Hebi City maintained a high water yield, followed by the vicinity of Junxian and Qixian. With the opening of the South-North Water Transfer Central Project, the water availability along both sides of the main trunk canal has increased significantly since 2015, especially near the recharge point of the Qi River, where the water availability has reached 630mm. The changes in water availability for the four periods 2000-2005, 2005-2010, 2010-2015 and 2015-2020 were calculated using tools such as the raster calculator in ArcGIS software to obtain Fig 9,and this figure is created using ArcMap 10.7, URL:wwwarcgis.com. It can be seen that in the two periods of 2000-2005 and 2005-2010, the main theme is the decrease of water yield, and the area of the decrease of water yield is 84.04% and 96.23% respectively; on the contrary, in the two periods of 2010-2015 and 2015-2020, the water yield continues to rise in most areas of Hebi city, and the area of the rise is 87.34% and 96.07% respectively. In general, the pattern of water availability in Hebi is strongly correlated with precipitation and vegetation evapotranspiration, with areas with high precipitation and low vegetation evapotranspiration usually maintaining a high level of water availability.

During the period 2000-2010, the overall water yield showed a decreasing trend, and the land use types in the areas where the water yield rose against the trend were mostly artificial land surface, while the overall water yield showed an increasing trend during the period 2010-2020, and there were still areas where the water yield decreased during the period, and the decreasing areas were sporadically distributed throughout Hebi City, and the land use types were mostly woodland, arable land and grassland. This shows that the water yield is closely related to the natural conditions such as precipitation and vegetation evaporation in the region, and the highest water yield is usually found in areas with high precipitation and low vegetation evaporation, which is consistent with the calculation principle of water yield.

### 4.4 Ecosystem service function zoning of Hebi City

**Fig 10:**
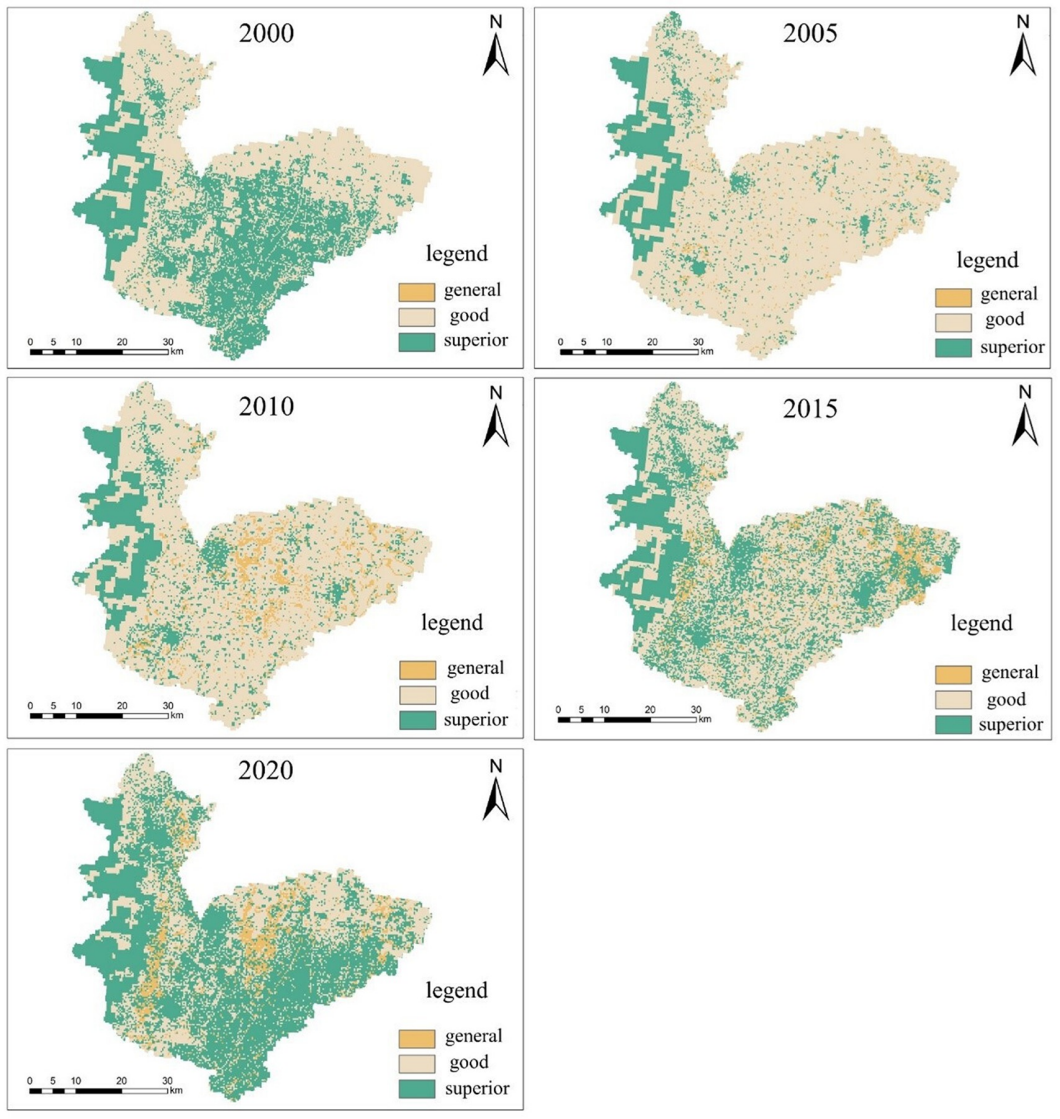
Comprehensive zoning map of ecosystem service functions in Hebi City over the years.

Combining the actual situation of Hebi city, habitat quality and water availability were organically combined, and the study idea of Zhao et al. [1] was referred to evaluate the zoning of ecosystem service function in the study area by three indicators of two ecosystem service functions. The spatial distribution of the three indicators was analyzed by overlaying them with ArcGIS software and processed with tools such as fusion, intersection, raster calculator, raster to surface, surface to raster, and reclassification to obtain the results of comprehensive zoning of ecosystem service functions in Hebi from 2000 to 2020, as shown in Fig 10, (this figure is created using ArcMap 10.7, URL:wwwarcgis.com) Table 14 details the area of different levels of ecosystem service functions between 2000 and 2020.

**Table 14:** Area distribution of ecosystem service levels in Hebi City between 2000 and 2020 (km^2^).

Grade	general	good	superior
2000	8.86	1166.62	1006.52
2005	51.52	1731.93	398.54
2010	156.47	1544.22	481.31
2015	148.88	1150.80	882.31
2020	187.59	742.84	1251.57

As can be seen from Fig 10, Hebi had a good ecological environment in 2000, and from 2000 to 2005, the ecosystem broke down more seriously, and the area of superior grade decreased seriously; after 2010, Hebi’s ecological environment improved significantly, and by 2020, Hebi’s ecological environment was basically restored. Comprehensive Fig 10 and Table 14 show that the overall development of integrated ecosystem service function in Hebi is positive. The area covered by the superior grade area continues to rise, and in 2020, the area covered by the superior grade area has exceeded that in 2000. The forested areas in the western mountainous areas of the city provide excellent ecosystem services. Different management plans are needed for different levels of ecosystem services to achieve better planning and management of ecosystem services, and the overall idea is to strictly implement and enforce environmental protection systems in areas with excellent and good service functions, and to increase improvements in soil and water conservation, water storage, urban greening and climate regulation in areas with average service functions.

### 4.5 Ecosystem service function drive analysis

In order to gain insight into the changes in ecosystem service functions and further study the extent of the impact of land use change and climate change on ecosystem service functions, this study examines the correlation between the landscape pattern index, precipitation and other data that are closely related to ecosystem service functions to drive the analysis of ecosystem service functions. A 5 km × 5 km grid was constructed as a spatial statistical unit in the study area, and only data grids without edge outliers were selected. 270 grid samples were constructed for five horizontal years. The meteorological factors, landscape pattern indices, habitat quality, habitat degradation indices, and water yield data sets of the 270 grid samples were analyzed by Pearson correlation analysis and two-tailed significance test using IBM SPSS Statistics 27 software.

#### 4.5.1 Correlation between landscape pattern indices and ecosystem service functions

The Hebi landscape pattern index dataset for 2000-2020 was correlated with the Hebi habitat quality, habitat degradation index and water yield dataset obtained from the InVEST model by SPSS27 software, and the results obtained are shown in Table 15.

**Table 15:** Pearson correlation analysis of ecosystem service function and landscape pattern index in Hebi City.

		LPI	NP	PD	ED	LSI	AI	CONTAG	SHDI
Habitat quality	Pearson Correlation	0.722	0.081	0.085	-0.914	-0.913	0.913	0.839	-0.777
	Significance (two-tailed)	0.169	0.898	0.892	0.030	0.030	0.030	0.076	0.122
Degree of habitat degradation	Pearson Correlation	-0.435	-0.235	-0.247	0.448	0.448	-0.443	-0.542	0.503
	Significance (two-tailed)	0.464	0.703	0.689	0.449	0.449	0.455	0.346	0.388
Water availability	Pearson Correlation	-0.448	-0.325	-0.318	0.229	0.230	-0.232	-0.303	0.345
	Significance (two-tailed)	0.449	0.593	0.602	0.711	0.710	0.708	0.621	0.570

The results of the study showed that the habitat quality in Hebi was positively correlated with the maximum patch index (LPI), number of patches (NP), patch density (PD), aggregation (AI) and spread (CONTAG), and negatively correlated with edge density (ED), landscape shape index (LSI) and Shannon diversity index (SHDI) during the period 2000-2020; Habitat quality index was significantly correlated with Edge density (ED), Landscape shape index (LSI) and Aggregation (AI), with the strongest correlation with Edge density (ED). The habitat degradation index is the opposite of habitat quality and is negatively correlated with the Largest patch index (LPI), Number of patches (NP), Patch density (PD), Aggregation (AI), and Contagion index (CONTAG), while it is positively correlated with Edge density (ED), Landscape shape index (LSI), and Shannon diversity index (SHDI); the strongest linear correlation with the degree of habitat degradation was spreading (CONTAG), with a Pearson correlation coefficient of -0.542, while the weakest was Number of patches (NP), with a Pearson correlation coefficient of only -0.235. Water yield was negatively correlated with Largest patch index (LPI), Number of patches (NP), Patch density (PD), Aggregation (AI), and Contagion index (CONTAG), while positively correlated with Edge density (ED), Landscape shape index (LSI), and Shannon diversity index (SHDI); the strongest linear correlation with water yield was the Largest patch index (LPI) with a Pearson correlation index of -0.448, while the weakest linear correlation was the Edge density (ED) with a Pearson correlation index of only 0.229.

**Fig 11:**
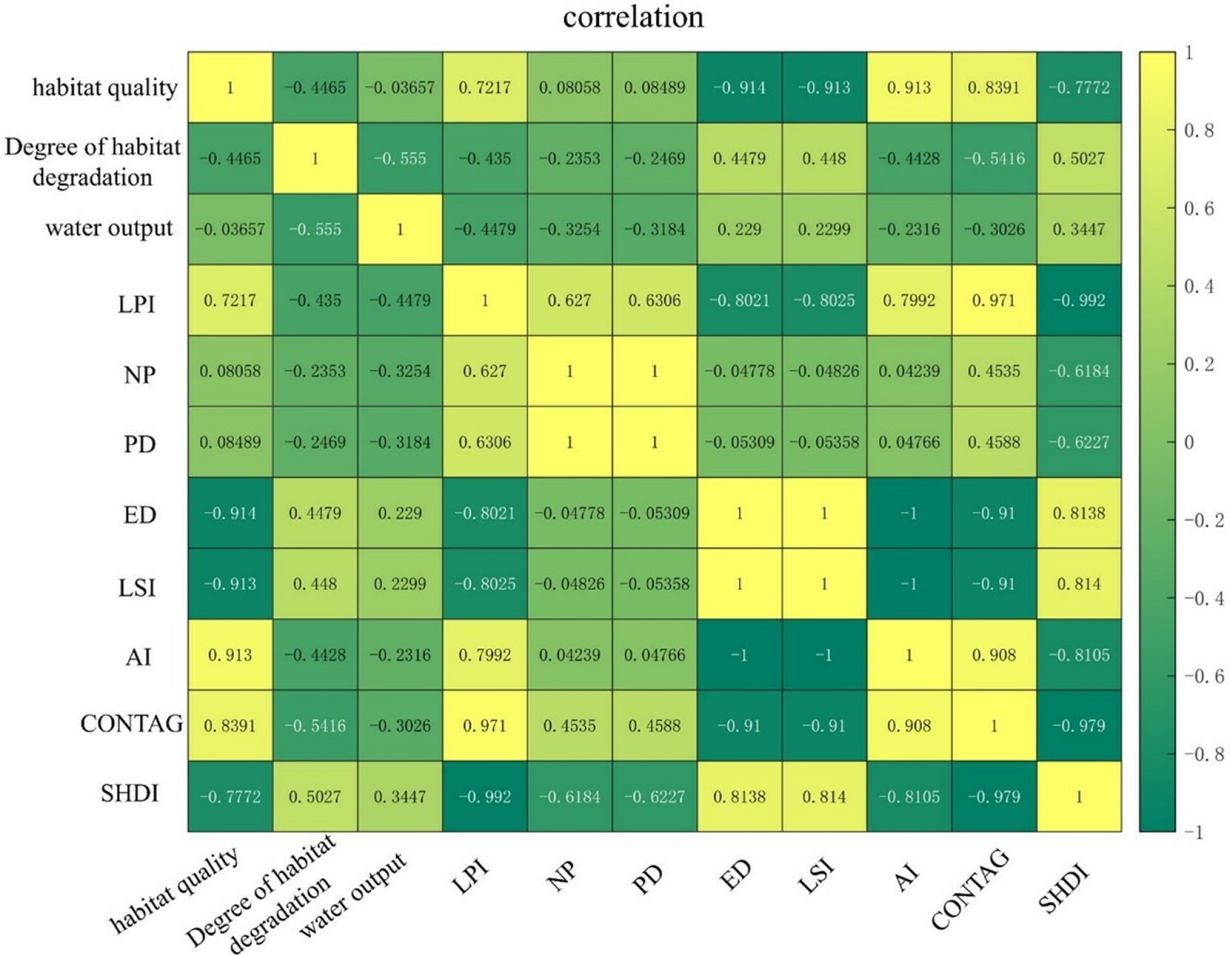
Heat map of correlation between ecosystem service function and landscape pattern index in Hebi City.

Overall, the ecological response of habitat quality to the landscape pattern index was the most pronounced among the ecosystem service functions in Hebi from 2000 to 2020, while the response of habitat degradation and water availability to the landscape pattern index was weak. The landscape pattern index, which is positively correlated with habitat quality, is negatively correlated with habitat degradation and water availability. The heat map of the correlation between ecosystem service function and landscape pattern index in Hebi is shown in Fig 11.

#### 4.5.2 Correlation between climate change and ecosystem services

To investigate the ecological effects of ecosystem response to climate change, this study correlated the precipitation dataset and the average annual temperature dataset of Hebi City from 2000 to 2020 with the habitat quality, habitat degradation index and water availability dataset of Hebi City obtained from the InVEST model by SPSS27 software, and the results obtained are shown in Table 16.

**Table 16:** Pearson correlation analysis between ecological environment service function and meteorological factors in Hebi City.

		Precipitation	Average annual temperature
Habitat quality	Pearson Correlation	0.169	0.325
	Significance (two-tailed)	0.786	0.569
Degree of habitat degradation	Pearson Correlation	-0.545	-0.447
	Significance (two-tailed)	0.342	0.451
Water availability	Pearson Correlation	0.961	0.335
	Significance (two-tailed)	0.009	0.049

The results of the study showed that the correlation between water yield and precipitation and average annual temperature in Hebi was significant during 2000-2020, and the correlation between water yield and precipitation was the strongest. The correlations between habitat quality and water availability and climate factors were positive, and the correlations between habitat degradation and climate factors were negative. Among the three types of ecosystem indicators, the response of habitat quality to annual mean temperature was higher than that of precipitation, the response of habitat degradation to precipitation was higher than that of annual mean temperature, and the Pearson correlation coefficient between water availability and precipitation reached 0.961.

## 5 Conclusion

1. From 2000 to 2020, the overall land use type of Hebi City keeps changing, gradually evolving from the initial type with the largest proportion of cultivated land area to the equal proportion of urban and cultivated land area. The change in transfer between land types is obvious, and the conversion of arable land to other land types is the most significant, with conversion to urban and grassland being the most common. The eight types of landscape level landscape pattern indices selected in the study all showed small changes between 2000 and 2020, and the five indicators LPI, NP, PD, AI, and CONTAG showed an overall increasing trend, in contrast to the three indicators ED, LSI, and SHDI, which showed an overall decreasing trend, indicating that the overall characteristics of significant landscape fragmentation and complexity exist in Hebi.
2. From the results of the habitat quality module assessment, the overall habitat quality in the study area showed a positive development trend. From a spatial perspective, the habitat quality of woodlands in Qixian County and the mountainous areas on the west side of Qibin District is the best, maintaining a superior grade throughout the study period, while the habitat quality grade in the bordering locations of Jun County and Qibin District gradually decreases due to various factors such as strong human activities, and most of the remaining areas show a trend of decreasing and then increasing habitat quality indices. In terms of land use types, the habitat quality index is best in areas where forest land exists, and the habitat quality index is lowest in areas where arable land and towns are densely distributed.
3. The overall water yield in the study area shows a trend of first decreasing and then increasing. The areas with high water yield are mainly concentrated in the westernmost part of Qibin District and the northwest part of Qixian County. In terms of land use types, the water yield is highest in the areas where large areas of forest land are gathered, and the water yield of urban and unused land types is generally higher than that of other land types except forest land.
4. The zoning of ecosystem services in Hebi was divided into three classes: superior, good and general. The results show that the area covered by the superior grade has been expanding since 2005; the area covered by the general grade has been increasing year by year with the influence of various factors such as urbanization and strong human activities.
5. The correlation analysis by SPSS software showed that climate change and landscape pattern index were the correlated factors causing changes in habitat quality and water availability in Hebi. The correlation between habitat quality and landscape pattern index is greater than that between habitat quality and climate change, and the strongest correlation with the landscape pattern index is edge density (ED); the correlation between water availability and climate change is greater than that between water availability and landscape pattern index, and the strongest correlation with the meteorological factor is precipitation.

## 6 Discussion

1. The scope of this paper is small and the findings may not be generalizable to other regions or other countries.
2. This paper only considered the impact of landscape pattern index and climate change on ecosystem service functions, and failed to take into account the impact of socio-economic changes, such as population density and gross regional product, on habitat quality, water yield and other functions in the study area. Later scholars can consider the influence of more comprehensive driving factors on regional ecosystem service functions.
3. In this study, we only assessed the habitat quality and water yield of the study area through the InVEST model, and did not analyze the results through multiple coupling analysis by combining other mechanistic models and statistical models. Subsequent researchers can utilize multiple coupled models to assess the regional ecosystem service function.

## Supporting information

S1 Data (DOCX)
