# Supplementary material for: Study of ecosystem service functions in typical receiving areas of the South-to-North Water Diversion Central Route based on a set of long time series
Source: PLoS One. 2024 May 15;19(5):e0302588. doi: 10.1371/journal.pone.0302588 (PMC11095674; doi:10.1371/journal.pone.0302588)
Supplement: S1 Data — (DOCX) [file pone.0302588.s001.docx]

| Data types | Data sources |
| --- | --- |
| DEM | Geospatial data cloud(http://www.gscloud.cn/) |
| Land use | Geospatial data cloud(http://www.gscloud.cn/) |
| Maximum root depth of the soil | World Soil Database(http://www.tpdc.ac.cn/zh-hans/ |
| Annual precipitation | National Earth System Science Data Center(http://www.geodata.cn/) |
| annual average potential evapotranspiration | the Consultative Group on International Agricultural Research of the Spatial Information Consortium(https://cgiarcsi.community/) |
